# Contribution to pulmonary diseases diagnostic from X-ray images using innovative deep learning models

**DOI:** 10.1016/j.heliyon.2024.e30308

**Published:** 2024-04-26

**Authors:** Akram Bennour, Najib Ben Aoun, Osamah Ibrahim Khalaf, Fahad Ghabban, Wing-Keung Wong, Sameer Algburi

**Affiliations:** a LAMIS Laboratiry, Echahid Cheikh Larbi Tebessi University, Tebessa, Algeria; b College of Computer Science and Information Technology, Al-Baha University, Al Baha, Saudi Arabia; c REGIM-Lab: Research Groups in Intelligent Machines, National School of Engineers of Sfax (ENIS), University of Sfax, Tunisia; d Department of Solar, Al-Nahrain Research Center for Renewable Energy, Al-Nahrain University, Jadriya, Baghdad, Iraq; e College of Computer Science and Engineering, Taibah University, Medina, Saudi Arabia; f Asia University, Taiwan; g Al-Kitab University, College of Engineering Techniques, Kirkuk, Iraq

**Keywords:** Pulmonary diseases diagnosis 1, Thoracic radiography, CXR images, COVID-19, Pneumonia, Pulmonary opacity, Deep learning

## Abstract

Pulmonary disease identification and characterization are among the most intriguing research topics of recent years since they require an accurate and prompt diagnosis. Although pulmonary radiography has helped in lung disease diagnosis, the interpretation of the radiographic image has always been a major concern for doctors and radiologists to reduce diagnosis errors. Due to their success in image classification and segmentation tasks, cutting-edge artificial intelligence techniques like machine learning (ML) and deep learning (DL) are widely encouraged to be applied in the field of diagnosing lung disorders and identifying them using medical images, particularly radiographic ones. For this end, the researchers are concurring to build systems based on these techniques in particular deep learning ones. In this paper, we proposed three deep-learning models that were trained to identify the presence of certain lung diseases using thoracic radiography. The first model, named “CovCXR-Net”, identifies the COVID-19 disease (two cases: COVID-19 or normal). The second model, named “MDCXR3-Net”, identifies the COVID-19 and pneumonia diseases (three cases: COVID-19, pneumonia, or normal), and the last model, named “MDCXR4-Net”, is destined to identify the COVID-19, pneumonia and the pulmonary opacity diseases (4 cases: COVID-19, pneumonia, pulmonary opacity or normal). These models have proven their superiority in comparison with the state-of-the-art models and reached an accuracy of 99,09 %, 97.74 %, and 90,37 % respectively with three benchmarks.

## 1 Introduction

Pulmonary diseases are disorders that affect the lungs and threaten their normal functioning. Pulmonary diseases can affect all people of the two sexes and all groups of age, including smokers and nonsmokers, which makes it one of the most widespread health issues. Thoracic radiography also referred to as chest X-ray or CXR, is a projective thoracic imaging used to diagnose a variety of diseases such as lung and cardiovascular diseases, the diseases affecting the bones of the rib cage or the spinal column, and cancers that originated in the region or spread there from other parts of the body [1]. Because of the high imaging quality it produces, thoracic radiography is frequently used by clinicians for diagnosis. This kind of examination is utilized to track the signs and medical problems in the chest region and helps in lung disease detection. Thoracic radiography is additionally utilized to monitor the effectiveness of the therapy. This makes it a good diagnostic source in addition to being readily available, non-invasive, and reasonably priced in comparison to other imageries [2].

Indeed, in radiographs of the thorax, several disorders, including pleurisy, pneumonia, bronchitis, infiltrations, nodules, heart hypertrophy, and others, can be seen as cavities, consolidations, infiltrations, widened coast angles, and nodules. This convergence of traits makes it challenging for radiologists to classify thoracic diseases using thorax radiography. However, in some extreme situations, it may be required to link a radiographic examination to other, more accurate and up-to-date examinations to save money and time, fill a specialist shortage, and/or aid in making an appropriate diagnosis [3]. Because of this, computer-assisted diagnosis (CAD) systems have been developed in recent years to aid physicians and radiologists in deciphering radiographs and gaining a clear understanding of the various disorders. Unfortunately, these CAD systems did not achieve the degree of significance required to establish pathological condition determinations on radiographs [4]. Consequently, they are kept just as a visualization tool that helps doctors and radiologists in making decisions. Recently, deep learning methods have demonstrated great success in the areas of feature extraction and image classification [[5], [6], [7], [8]]. When carrying out these tasks, various cutting-edge deep networks demonstrate an extraordinary level of precision which inspired researchers to use them for the classification of medical images [[9], [10], [11], [12]]. The findings demonstrated that deep networks are capable of successfully extracting important traits that distinguish different classes of diseases through the automatic analyses of medical images [13]. The convolutional neural network (CNN) is one of the most well-reputed network types that was frequently utilized for the analysis and extraction of characteristics from various types of images. In the landscape of medical imaging, advancements in deep learning techniques have revolutionized disease diagnosis, particularly in the realm of pulmonary infections. Despite significant strides, there remains a crucial need for novel contributions that push the boundaries of existing research. In this study, we aim to bridge this gap by presenting innovative approaches in pulmonary disease classification based on chest X-ray (CXR) images. Our work builds upon the latest advancements in deep learning architectures, focusing on the efficient and accurate identification of COVID-19, pneumonia, pulmonary opacity, and normal lung cases. By leveraging state-of-the-art methodologies and datasets, we introduce novel models tailored to address the complexities and challenges inherent in diagnosing pulmonary infections from CXR images. Through rigorous experimentation and evaluation, we demonstrate the superior performance and robustness of our proposed models, highlighting their potential to significantly impact clinical practice and patient outcomes. In this paper, CNN-based models for the recognition of pulmonary diseases from radiological images are suggested. Three deep learning models have been developed and achieved high accuracy in the automated detection of several pulmonary diseases, including COVID-19, pneumonia, and pulmonary opacity from chest X-ray images, which will reduce the workload of radiologists and enhance the speed and precision of diagnosis. These models are designed to deal with various classification scenarios, ranging from binary (COVID-19/non-COVID-19) to four-class (COVID-19, pneumonia, pulmonary opacity, normal) classification. Each model is tailored to the specific classification problem it addresses and has distinct architectural features that reflect the nature of the problem and data. A comparative study with the state-of-the-art method evaluated on the same datasets has confirmed the efficiency of the proposed models.

The remainder of this paper is structured as follows. In the next section, the recent related works will be presented while providing their results which will be used to compare them later with our work. Afterward, section 3 details the proposed deep learning-based pulmonary disease identification models. Furthermore, in section 4, an experimental study is provided with the datasets used for evaluation, the experimental results of our models as well as a comparative study with the state-of-the-art methods to demonstrate the efficiency and the usefulness of the proposed architectures in the field of pulmonary diseases detection, especially the COVID-19. Finally, the conclusion section summarizes the key findings and suggests future potential research directions.

## 2 Related works

The accurate diagnosis of pulmonary diseases, especially COVID-19, has become increasingly vital since the onset of the pandemic. Leveraging deep learning techniques for detecting and classifying pulmonary diseases using X-ray images has been the focus of several studies, aiming to aid medical professionals in accurate and timely diagnosis.

Tripathi et al. [14] proposed a deep convolutional neural network architecture achieving an average disease classification precision of 89.77 % using the “Chest X-Ray 14″ dataset. Similarly, Ozturk et al. [15] explored binary and multi-class classification approaches, achieving notable classification precision rates for distinguishing COVID-19 cases from other lung diseases. Furthermore, Wong et al. [16] introduced COVID-Net, specifically designed for COVID-19 detection with an accuracy of 93.3 %, while Mudasir et al. [17] proposed CoroNet, achieving high precision rates for various classification scenarios. Various other models such as CovXNet [18], CVDNet [19], Deep CNN [20], CapsNet [21], and COVIDiagnosis-Net [22] have also contributed with unique approaches to pulmonary disease diagnosis, showcasing precision rates ranging from 84.22 % to 98.26 %.

Recent studies have explored diverse deep-learning approaches for COVID-19 and pulmonary disease classification using thoracic radiography. For instance, researchers [23] developed a deep learning system achieving high accuracy rates for classifying COVID-19, pneumonia, and normal cases. Another investigation [24] assessed the performance of deep network architectures, obtaining high classification precision and sensitivity. In a separate study [25], DeepCCXR models achieved impressive accuracies for binary and multi-class classification of pulmonary diseases, utilizing multiple datasets including COVID-19, Image Dated Collection (CIDC) [26], COVID-19 Radiography [27], RSNA [28], Chest X-Ray Images (Pneumonia) [29], Montgomery County X-ray [30], Shenzhen Hospital X-ray [31], and Montfort Dataset [32], they trained and tested their models. Based on the fine-tuning of an EfficientNet-B5 architecture, these models achieved an impressive accuracy of 93 % for multi-class classification (COVID-19/normal/pneumonia) and 96 % for the binary model (COVID-19 vs normal). The average sensitivity was reported as 97 % and 94 %, respectively.

Furthermore, novel approaches such as stacked ensemble models [33] and algorithm optimizations [34] have been explored to enhance disease diagnosis accuracy. Additionally, Bharati et al. introduced CO-IRv2 [35] and Bayesian optimization-based CNN models [36] for COVID-19 diagnosis, demonstrating remarkable accuracies and sensitivities. Other investigations proposed various deep learning models [37], achieving accuracies between 94.5 % and 99.55 %.

These studies collectively contribute to improving the accuracy and reliability of COVID-19 and pulmonary disease diagnosis using deep learning techniques applied to thoracic radiography. Further, Recent advancements in chest X-ray identification highlight the potential of deep learning in enhancing diagnostic accuracy and efficiency, such as Shammi et al. [38] who proposed a MobileNetV2-based approach achieving an average accuracy of 98.65 % for distinguishing normal pneumonia or COVID-19 pneumonia conditions. Chamodi et al. [39] conducted a comparative study employing multiple deep learning architectures, including ResNet50, achieving an average accuracy and recall of 98.87 % and 98.54 %, respectively, for detecting normal, pneumonia, and COVID-19 conditions. From their side, Kumarasinghe et al. [40] introduced a modified U-Net architecture for chest X-ray segmentation, achieving an Intersection over Union of 93.53 % and 99.83 % accuracy on segmentation-aided ensemble classification for COVID-19 and pneumonia. Additionally, Dulani et al. [41] conducted a systematic review highlighting the state-of-the-art deep learning-based solutions for pneumonia and COVID-19 detection from chest X-ray images, providing insights into recent trends, datasets, challenges, and future research directions. More recently, Wimukthi et al. [42] proposed MERGIS, a transformer-based encoder-decoder model leveraging image segmentation to improve automatic medical report generation for chest X-rays, outperforming current state-of-the-art models with enhanced accuracy scores. These studies collectively illustrate the growing potential of deep learning techniques in enhancing the accuracy and efficiency of chest X-ray diagnosis, paving the way for improved healthcare outcomes. Table 1 provides a summary of the most accurate works cited above for better visualization and comparisons.

**Table 1:** Summary of the related works.

Model		Dataset	Classes	Accuracy (%)	Sensitivity (%)
**DarkCovidNet** [15]		CIDC ChestX-ray14 dataset	3	87.02	85.35 %
**MobileNet-v2** [15]			4	86.6	98.4
**CNN** **+** **VGG** **+** **STN** [14]		Chest X-Ray 14	3	89.77	/
**COVID-Net** [16]		COVIDx dataset	3	93.3	/
**CoroNet** [17]		CIDC, RSNA	4	89.6	96.4
			3	95	97.5
			2	99	98.6
**CovXNet** [18]		Collected Data	4	90.1	89.3
			3	94.7	93.3
			2	97.4	96.9
**CVDNet** [19]		COVID-19 Radiography Database	2	97.20	/
			3	96.69	/
**Deep CNN** [20]		CXR dataset	2	95.2	96.0
			4	78.80	89.70
**CapsNet** [21]		CIDC	3	84.22	/
			2	97.24	/
**Deep-Covid** [43]	ResNet18	COVID-19 Radiography Database, ChexPert dataset	2	98.9	98
	ResNet50		2	99.0	98
	SqueezeNet		2	99.2	98
	DenseNet-121		2	97.6	98
**EfficientNet** [44]		CIDC, RSNA COVIDx dataset, HCV-UFPR COVID-19 dataset	2	93.9	96.8
**COVIDiagnosis-Net** [22]		COVIDx	3	98.26	98.26
**Proposed CNN** [23]		CIDC	2	97.62	95
			3	96.78	95.43
**VGG19** [24]		CIDC, RSNA COVIDx dataset	2	98.75	92.85
**VGG16** [24]			3	83.5	94.43
**DeepCCXR** [25]		9 datasets	2	96	97
			3	93	94
**Stacked collaboration of VGG-19 and DenseNet-169 models** [33]		Locally collected data-set	2	93	93
**CO-ResNet: Optimized ResNet101** [34]		COXECOVID dataset	3	98.99	90
**CO-IRv2: derived from the InceptionNet and ResNetV2 methods** [35]		COVID-CT dataset	2	99.40	99.38
**Bayesian optimization-based convolutional neural network (CNN)** [36]		OpenI dataset	2	96 %	/
**ResNet152V2** [37]		Collected dataset	2	95.74	/
**LDDNet: Optimized DenseNet201** [37]				99.55	/
**XceptionNet** [37]				94,5	/
**MobileNetV2-based** [38]		Collected dataset	2	98.65 %	98 %
**ResNet50** [39]		Collected dataset	2	98.87	98.54
**U-Net architecture** [40]		Collected dataset	2	93.53 %	93 %

## 3 Adopted approaches

In this section, we will detail the different approaches used in this work, our proposed CNN architectures, and the mildly fine-tuned VGG16 models with preloaded ImageNet weights from data preparation to the validation step.

### 3.1 Bi-class classification model (CovCXR-Net)

CovCXR-Net (COVID-19 Chest X-rays Network) is the first proposed model for diagnosing COVID-19 disease using Chest X-ray images. It is novel in its simplicity, tailored specifically for the task, and designed to maximize the use of available data. Previous works have used more complex architectures such as deep residual networks, which are not always efficient especially due to the limited amount of data available. Several studies on diagnosing pulmonary diseases using X-ray images also found that smaller CNN models with fewer layers and parameters can perform comparably to larger models while being computationally efficient, [45]. The proposed model consists of two blocks, each of which has two convolution layers (3 × 3), two Maxpooling layers (2 × 2), and a dropout of 25 %.

This choice was motivated by several key considerations. Firstly, empirical experimentation and hyperparameter tuning were conducted to identify the dropout rate that yielded optimal performance on the small-size dataset and the need for a computationally efficient model. Through iterative testing, it was observed that a dropout rate of 25 % effectively regularized the model, reducing overfitting while preserving the model's capacity to learn complex features from the data [46]. Furthermore, prior research in the field of deep learning and medical image analysis has suggested that moderate dropout rates, typically ranging from 20 % to 50 %, are effective in preventing overfitting in Bi-classifier convolutional neural networks (CNNs) trained on small datasets. By adhering to this empirical guideline, we aimed to strike an effective balance between bias and variance, ensuring that the CovCXR-Net model generalizes well to unseen data while maximizing its diagnostic accuracy.

The image in the entry, of size 128 × 128 × 3, is injected initially into the first convolution layer. This layer is made up of 32 filters of size 3 × 3, each one of the convolution layers is followed by an activation function ReLU. This function forces the neurons to turn over positive values. As a result, 32 feature maps of size 128 × 128 are generated. Afterward, we apply max-pooling of size 2 × 2 with a stride of two to reduce the size of the image and the number of parameters thus the calculation time which will result in obtaining 32 feature maps. These feature maps were transmitted to the following block. The same scenario is held in the other blocks with an increase in the number of filters (as explained previously) until reaching the last max-pooling layer which creates 64 feature maps of size 8 × 8.

**Fig. 1:**
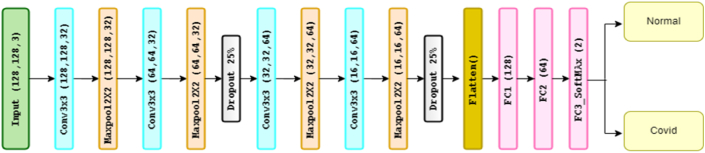
CovCXR-Net architecture.

After the flattened layer, we used three entirely connected layers; the first layer is made up of 64 neurons followed by a layer of 32 neurons with ReLU activation. Then, the last layer is made up of 2 neurons with a Softmax activation function that gives the bi-classes distribution probability (see Fig. 1). The use of dropout was also chosen to prevent overfitting by randomly dropping out some of the neurons during training. This technique is effective in preventing overfitting in deep learning models. Using the ReLU activation function after each convolution layer was encouraged by its effectiveness in enhancing the model's ability to learn complex features. It was made to introduce non-linearity in the model and to avoid the vanishing gradient problem. In addition, it is also common in CNNs and has been shown to work well in many applications [47].

The choice of a 128 × 128 input size and max-pooling layers with a stride of 2 has been made to reduce the size of the image and the number of parameters. This leads to a reduction in the spatial dimension of the feature maps and introduces translation invariance in the model, thus improving computational efficiency while retaining sufficient information for accurate diagnosis [48].

In conclusion, the first proposed model for diagnosing pulmonary diseases using X-ray images is novel in its simplicity and tailored design. The choice of a small CNN architecture with dropout and ReLU activation functions, small input sizes, and pooling layers is motivated by the small size of the dataset and the need for computational efficiency. The model provides accurate diagnosis while using a minimal number of parameters, making it a computationally efficient and practical solution for diagnosing COVID-19 using X-ray images.

### 3.2 Multi-class classification models

Compared to the bi-class disease classification model previously presented, multi-class disease classification, using a single deep network, requires more complex architectures. As the number of classes increases, the decision boundary between them becomes more complex, and a more sophisticated model is required to capture the nuances of the data [49]. As mentioned in the previous section, in the context of X-ray image analysis, more complex models have been shown to improve the performance for multi-class classification tasks. For example, recent studies [50] have shown that using deeper CNN architectures such as DenseNet or ResNet can improve accuracy on pulmonary diagnosis tasks while other research works [49,51] have proved that transfer learning from pre-trained models such as VGG (Visual Geometry Group) or Inception can also improve performance on small datasets by leveraging the learned features from larger datasets.

In this work, to address the multi-class pulmonary diseases classification, we have followed a transfer learning approach by using a pre-trained model based on the VGG16 which is a common and effective practice in deep learning, particularly when working with limited data in medical images analysis [52]. Indeed, pre-trained models have been trained on large datasets, such as ImageNet, which allows them to capture general features and patterns, and learn generic features that can be fine-tuned on a smaller dataset and be very useful for a wide range of computer vision tasks and achieve high classification and accuracy. Indeed, VGG16 has been used as the backbone model while fine-tuning only the last layer. Indeed, the choice of VGG16 was supported by its effectiveness in the ImageNet challenge and its popularity in the computer vision community [53,54]. In addition, the availability of pre-trained weights for VGG16 makes it easy to implement and fine-tune our model for specific tasks. Additionally, in VGG16, the lower layers of the network learn low-level features that are likely to be useful for a variety of tasks, while the higher layers learn more task-specific features. By freezing the lower layers and fine-tuning only the last layer, the pre-trained network has been adapted to our specific task while still leveraging the useful features learned in the lower layers. Although pre-trained models have been used in previous studies for pulmonary disease diagnosis, our proposed models are built in a pre-trained model while enhanced with additional architectural tuning at the last level of the network to accurately identify the pulmonary diseases from the treated dataset. Two models have been built: MDCXR3-net which tends to identify three diseases (COVID-19, pneumonia, and normal), and MDCXR4-net which tends to identify four diseases (COVID-19, pneumonia, pulmonary opacity, and normal).

#### 3.2.1 MIDCXR3-net model

**Fig. 2:**
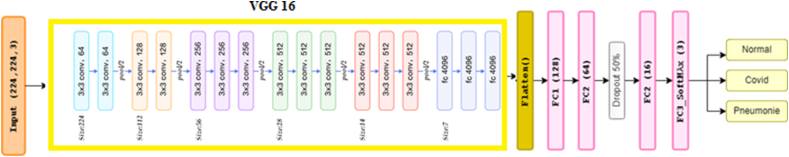
MIDCXR3-net architecture.

MDCXR3-Net (Multi-Diseases Chest X-rays Network) model is devoted to classifying three pulmonary diseases (COVID-19, pneumonia, and normal), the weights of the VGG16 pre-trained model on the “ImageNet” dataset have initiated our network while a flattened layer and 4 entirely connected layers have been added. The first fully connected is composed of 128 neurons, followed by a layer that comprises 64 neurons and a dropout of 50 %, then a layer of 16 neurons. Then, the last layer contains 3 neurons with Softmax as an activation function (see Fig. 2).

The choice of a dropout rate of 50 % was guided by several key considerations. Firstly, empirical experimentation and hyperparameter tuning were conducted to identify the dropout rate that yielded optimal performance on our dataset. Through iterative testing, we observed that a dropout rate of 50 % effectively regularized the model, mitigating overfitting while preserving the model's ability to learn complex features from the data. By adhering to this empirical guideline, we aimed to strike an effective balance between bias and variance, ensuring that the MIDCXR3-Net model generalizes well to unseen data while maximizing its diagnostic accuracy. Additionally, the choice of a dropout rate of 50 % aligns with the principle of model regularization, which seeks to prevent the model from memorizing noise in the training data and encourages it to learn robust and generalizable features. By introducing dropout at a higher rate, we aimed to increase the model's resilience to noisy or irrelevant information in the training data, thereby enhancing its ability to generalize to unseen data and improve diagnostic accuracy.

#### 3.2.2 MIDCXR4-net model

**Fig. 3:**
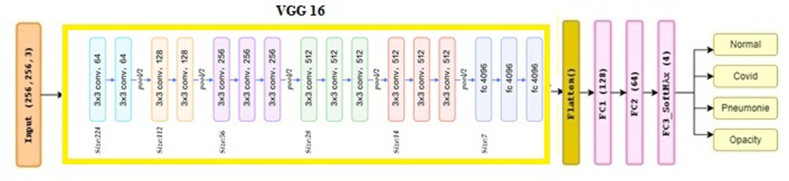
MDCXR4-Net architecture.

MDCXR4-Net model is developed to classify four pulmonary diseases (COVID-19, pneumonia, pulmonary opacity, and normal). In addition to the pre-trained VGG16 which is considered as the model backbone, 3 fully connected layers have been added. The first layer is formed of 128 neurons, the second layer has 64 neurons, and the last one is composed of 4 neurons with a Softmax activation function (see Fig. 3).

Actually, for the two MDCXR3-Net and MDCXR4-Net, the VGG16 model has been fine-tuned in each model to conduct a classification into 3 and 4 diseases classes instead of the original 1000 classes it was trained on in the ImageNet dataset. This modification has required an adjustment of the last layer and retraining of only a small portion of the network, which has saved time and computational resources while achieving good results. In addition, our strategy of fine-tuning only the last layer of the pre-trained model, rather than retaining the entire network has experimentally shown its effectiveness. Furthermore, adopting a single deep network approach for the classification tasks has several advantages compared to a model based on the combined networks since it is computationally, requires less memory and computational power, and is beneficial when working with large datasets or deploying the model in resource-limited environments. Additionally, a single deep network allows for end-to-end learning, eliminating the need for handcrafted feature engineering and reducing the risk of information loss during feature extraction. Also, using a single deep network can lead to better performance compared to using voting or combination techniques and can be easier to interpret and provide insights into the underlying patterns in the data [55].

## 4 Experimentation study

This section provides the experimental environment: model parameters, datasets, and the model performance evaluation metrics. In addition, the experimental results of the proposed models will be presented as well as a comparative study with the state-of-the-art methods previously reported.

All training was done in the Google Colaboratory execution environment, which provides a Tesla T4 GPU or equivalent with 16 GB of VRAM, 2 threads of an Intel Xeon CPU with 12–13 GB of system RAM, and approximately 41 GB of cloud disk storage available for 12 h a day. However, for the brief instances where we had to use a local execution environment, we opted to use Anaconda Navigator (anaconda3) version 3.9.7 with Jupiter Notebook version 6.4.8. The TensorFlow and Keras versions in this case were 2.6.0. Each model was trained using the presented details in Table 2.

**Table 2:** Training parameters.

Model	Patch size	epoch	Stage per epoch
CovCXR-Net	32	60	7
MDCXR3-Net	32	20	20
MDCXR4-Net	32	10	133

### 4.1 Datasets

In our study, we used three datasets (See Table 3): COVID-19 Image Dated Collection (CIDC) [56], Chest X-Ray Images Pneumonia (CXRP) [57,58] and COVID-19 Radiography database (CRD) [59,60]. CIDC was created by Dr. Joseph Paul Cohen [55] and is made up of CXR and CT scan images of patients infected with COVID-19 and other pneumonia information was mainly extracted from medical Websites that collected images of CXR of COVID-19 publicly available from hospitals and clinicians. The dataset includes 654 images of CXR COVID-19 coming from various sources. Whereas the CXRP dataset is composed of 5863 images of radiography for 2 categories: pneumonia and normal case. The images of radiography of the thorax were selected starting from retrospective troops of old pediatric patients of one to five years of Guangzhou Women and Children’s Medical Center. All radiographies of the thorax were carried out within the framework of the clinical care of routine of the patients. For the analysis of the pulmonary radiographic images, all pulmonary radiographic initially was the quality control object by eliminating all the scans from bad quality or illegible. The diagnoses of the images were then evaluated by two medical experts before being authorized to involve any IA system. To take account of possible errors of classification, the whole evaluation was also checked by a third expert [57,58]. In addition, the CRD dataset was created by a research team of the University of Qatar and the University of Dhaka (Bangladesh) in collaboration with doctors. In the first version of this dataset [59], the creators of the database published 219 images of pulmonary radiography of COVID-19, 1341 images of normal, and 1345 images of viral pneumonia. Then the dataset has been updated [60] by increasing the image number to reach 1200 CXR images with COVID-19 infection. A second update has been done to increase the dataset with 3616 positive cases of COVID-19, 10192 normal images, 6012 pulmonary opacities, and 1345 images of viral pneumonia.

**Table 3:** Summary of the used datasets.

Dataset	Class	Total Images
**CIDC** [56]	COVID-19	654
**CXRP** [57,58]	Pneumonia	5863
	Normal Lung	
**CRD** [59,60]	COVID-19	3616
	Normal Lung	10192
	Pulmonary Opacity	6012
	Viral Pneumonia	1345

Based on these three datasets, we have created our dataset to experimentally evaluate our models. For the first CovCXR-Net model, 145 COVID-19 CXR images were taken from the CIDC dataset, and 140 images of the normal lung from the CXRP dataset. Then, the resulting dataset (285 images) was split into around 80 % for model training (225 images) and around 20 % for model testing (60 images). To evaluate the MDCXR3-Net model, a dataset is constructed with 648 COVID-19 images and 648 images with pneumonia infections from the CIDC dataset as well as 648 normal lung images from the CXRP dataset. Then, from the resulting 1944 images, 1500 images (around 80 %) were used to train the model while 444 images (around 20 %) were used to test it. However, to evaluate the MDCXR4-Net mode, 1000 images for each class (COVID-19, pneumonia, pulmonary opacity, and normal lung) have been collected from the CRD dataset. As previously done, around 80 % of the dataset has been used to train the model (2940 images) and around 20 % of the dataset has been used for testing (1060 images).

Table 4 below highlights the number of used images in each class in the dataset, and State the split ratio.

**Table 4:** Used dataset details.

Model	Class	Total Images	Training Images	Testing Images	Split Ratio %
**CovCXR-Net**	COVID-19	145	116	29	80/20
	Normal Lung	140	112	28	80/20
**MDCXR3-Net**	COVID-19	648	500	148	78/22
	Pneumonia	648	500	148	78/22
	Normal Lung	648	500	148	78/22
**MDCXR4-Net**	COVID-19	1000	735	265	74/26
	Pneumonia	1000	735	265	74/26
	Pulmonary Opacity	1000	735	265	74/26
	Normal Lung	1000	735	265	74/26

### 4.2 Models performance evaluation metrics

**Fig. 4:**
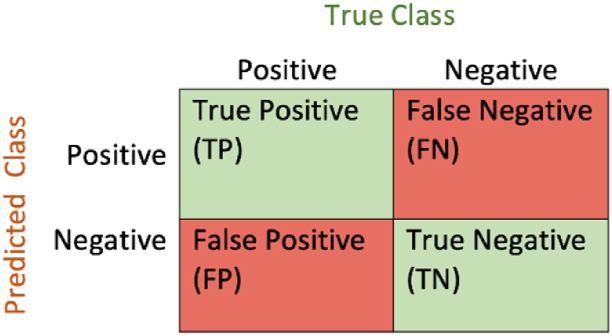
Confusion matrix Where True Positive (TP) and True Negative (TN) refer to the number of correctly predicted positive and negative samples while the False Positive (TP) and False Negative (TN) refer to the incorrectly predicted samples respectively.

To evaluate the proposed models, we referred to the confusion matrix which is a table used to visualize and summarize the performances of the classification algorithms [61]. Each column of the matrix represents the instances of a real/true class, while each line represents the predicted ones as shown in Fig. 4.

In addition to the confusion matrix, different classification model evaluation metrics have been employed such as the accuracy, the precision, the recall, and the F1 score, which are calculated based on values TP, TN, FP, and FN previously mentioned as follows:

- ***Accuracy (ACC)***: give the proportion of the full number of correct predictions:

(1) Accuracy=(TP+TN)/(TP+TN+FP+FN)

- ***Precision***: or the positive predictive value (PPV), is the proportion of positive values compared to the total of the predicted positive instances. In other words, the precision is the proportion of positive values that were correctly identified:

(2) Precision=TP/(TP+FP)

- ***Recall***: also called sensitivity or true positive rate (TPR) is the proportion of positive values compared to the total of the real positive cases:

(3) Sensitivity=TP/(TP+FN)

- ***F1 score*** is the harmonic mean of the precision and the recall:

(4) F1score=2×(Precision×Sensitivity)/(Precison+Sensitivity)

### 4.3 Experimental results

**Fig. 5:**
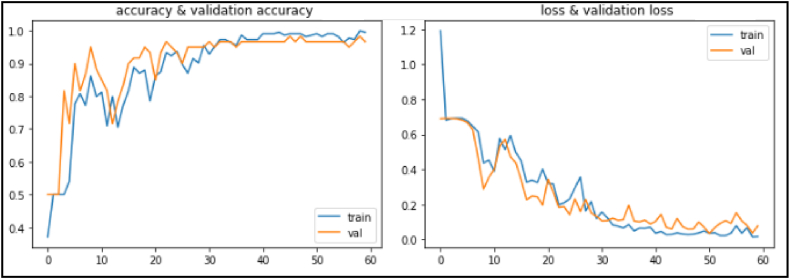
Training/validation accuracy and loss for the CovCXR-Net model.

**Fig. 6:**
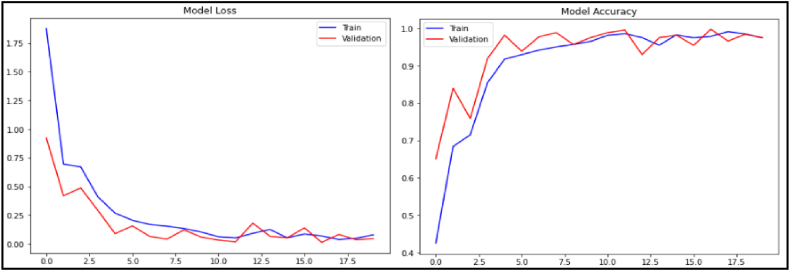
Training/validation accuracy and loss for the MDCXR3-Net model.

**Fig. 7:**
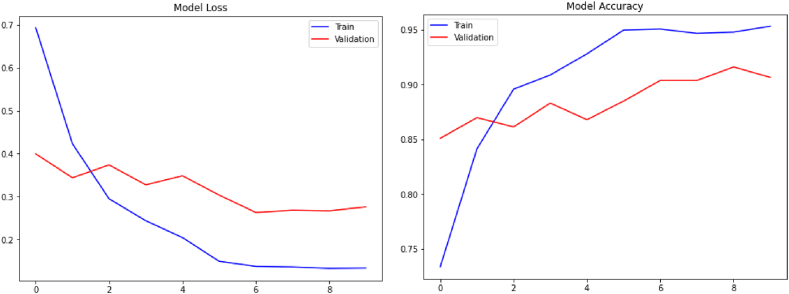
Training/validation accuracy and loss for the MDCXR4-Net model.

Evaluated on the aforementioned datasets, the three developed methods have given very promising results. As illustrated in Fig. 5, Fig. 6, Fig. 7, the performances of the proposed models (accuracy and loss) during the training and validation phases are highlighted. These figures clearly show the convergence of our three models in terms of accuracy maximization and loss function minimization during both the training and the validation phases, which prove the good performance of our models. In addition, after some fluctuations in the middle of the experiments which can be explained by the low quality of some images in the datasets, the models attempt to the stabilization status by the last training/validation epochs to reach the maximum classification accuracy and the minimum loss.

**Fig. 8:**
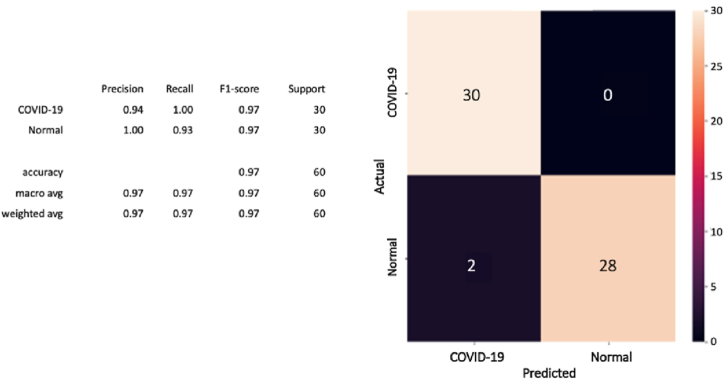
The confusion matrix of the CovCXR-Net on the validation data.

**Fig. 9:**
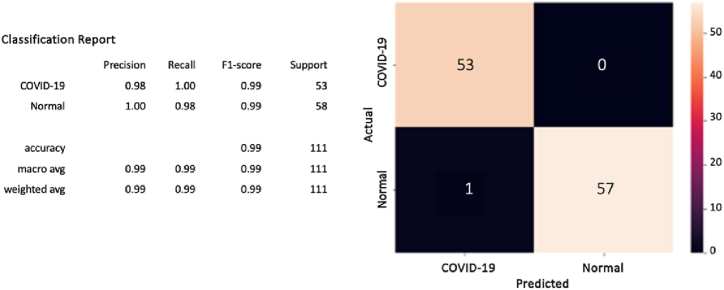
The confusion matrix of the CovCXR-Net on the test data.

**Fig. 10:**
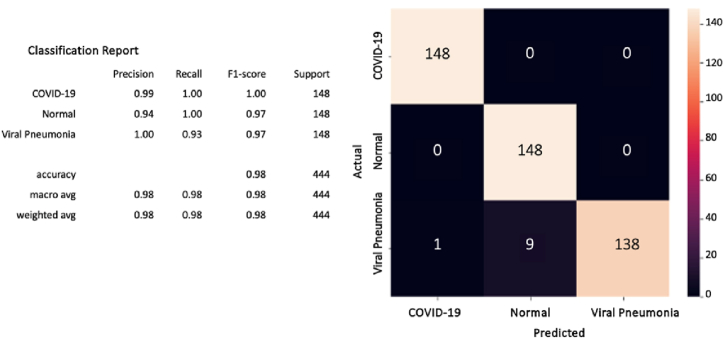
The confusion matrix of the MDCXR3-Net on the test data.

Furthermore, after computing the confusion matrix of each model (see Fig. 8, Fig. 9, Fig. 10), equation (1) (2) (3), and (4) have been applied to calculate the accuracy, precision, recall/sensitivity, and F1-score to measure the performance for each model.

#### 4.3.1 CovCXR-net model

The confusion matrix shown in Fig. 8 presents the experimental results for the CovCXR-Net model on the validation data (60 images). It illustrates that the model has correctly detected all the positive COVID-19 cases.

To confirm the performances of the model, it has been additionally tested on test data on which he had not learned yet. The test data containing 111 images was divided into 58 normal and 53 COVID-19. Fig. 9 below provides the obtained results in which the CovCXR-Net model has predicted all the cases except one normal case that has been mistakenly identified as a COVID-19 case.

#### 4.3.2 MDCXR3-net model

Similarly, after it has been learned, the MDCXR-Net model is tested on 444 images (148 images of COVID-19 cases, 148 images of pneumonia cases, and 148 images of normal lungs). As shown by the confusion matrix results, the model performed well and detected all the COVID-19 and pneumonia cases (see Fig. 10).

#### 4.3.3 MDCXR4-net model

**Fig. 11:**
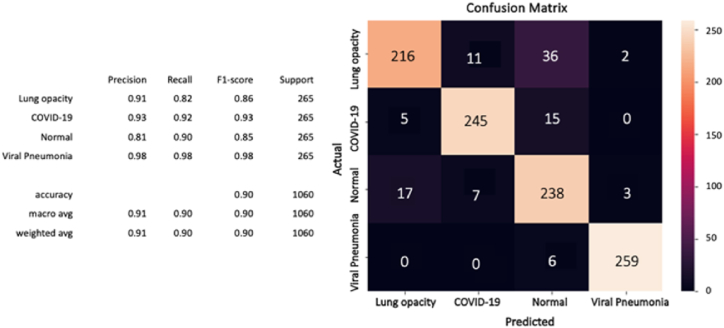
The confusion matrix of the MDCXR4-Net on the test data.

In a similar way as previously, MDCXR4-Net has been tested on 1060 images (265 of each of the four classes). It can be noticed from Fig. 11 that the model has identified well the diseases and normal cases especially the viral pneumonia and the COVID-19. While many lung opacity cases have been detected as normal which can be explained by the bad quality of some images where the opacity is not sufficiently clear.

More evaluations have been made to measure the three models’ performances (see Table 5). The obtained results confirm the effectiveness of our model. Indeed, as can be seen from Table 3, increasing the number of pulmonary diseases to be diagnosed will make the model more confused and result in a decrease in the model performance. So, it is encouraged to tailor the model to the minimum number of diseases when possible.

**Table 5:** Models’ performances in terms of accuracy, precision, sensitivity, and F1-score.

Model	Accuracy (%)	Precision (%)	Sensitivity (%)	F1-score (%)
CovCXR-Net	99.09	99.07	99.13	99.09
MDCXR3-Net	97.74	97.86	97.74	97.79
MDCXR4-Net	90.37	90.67	90.37	90.51

### 4.4 Comparison with the state-of-the-art methods

Table 6 showcases a comparison of the bi-class proposed model with similar works from the literature. As it can be noticed, our proposed model outperformed existing state-of-the-art methods in terms of accuracy and sensitivity which indicates that our model is better at correctly identifying positive cases and minimizing false positives. Specifically, our model achieved an accuracy of 99.09 %, which is higher than the other presented methods.

**Table 6:** Performance comparison of the CovCXR-Net model with similar works.

Model	Dataset	Classes	Accuracy (%)	Sensitivity (%) of Covid-19
CoroNet [17]	CIDC, RSNA	2	99	98.6
Deep CNN [20]	CXR dataset	2	95.2	96.0
EfficientNet [44]	CIDC, RSNA COVIDx dataset, HCV-UFPR COVID-19 dataset	2	93.9	96,8
CNN [23]	CIDC	2	97.62	95,43
VGG19 [24]	CIDC, RSNA COVIDx dataset	2	98.75	92.85
DeepCCXR [25]	Multiple datasets	2	96	97
	CIDC	2	91	91
	RNSA	2	98	99
DNN [53]	Chest X-Ray14	2	90.10	89.00
DenseNet-121 [62]	CIDC	2	93.00	93.80
Inception V3 [16]	CIDC, RSNA	2	98.17	91.45
ResNet-50 [16]	CIDC, RSNA	2	98.50	93.30
**Our Model (CovCXR-Net)**	**CIDC, Chest x-ray Pneumonia**	**2**	**99.09**	**99.13**

Indeed, in comparison with the other state-of-the-art models, CovCXR-Net has a smaller architecture and fewer parameters targeting the COVID-19 classification task which leads to better computational efficiency without sacrificing accuracy. While other reported models like CoroNet, VGG19, Inception V3, and ResNet-50 provide high accuracies, they are more complex than CovCXR-Net. So, the proposed CovCXR-Net model strikes a balance between model complexity and accuracy, making it a practical solution for diagnosing pneumonia using X-ray images.

Furthermore, MDCXR3-Net model performances have been compared to the state-of-the-art methods as illustrated in Table 7. The proposed model succeeded in effectively identifying two diseases (COVID-19 and pneumonia) and outperformed the majority of the methods except for COVIDiagnosis-Net where it was comparable. The proposed model achieved an accuracy of 97.74 % with a 97.79 % sensitivity which proves the model's strength.

**Table 7:** Performance comparison of MDCXR3-Net model with similar works.

Model	Dataset	# Classes	Accuracy (%)	Sensitivity (%)
DarkCovidNet [15]	CIDC	3	87.02	87.02
CoroNet [17]	CIDC, RSNA	3	95	97.5
CVDNet [19]	COV- RD	3	96.69	94.3
CapsNet [21]	CIDC	3	84.22	/
COVIDiagnosis-Net [22]	COVIDx	3	98.26	98.26
CNN [23]	CIDC	3	96.78	95.43
VGG16 [24]	CIDC, RSNA COVIDx dataset	3	83.5	94.43
InceptionV3 [63]	CIDC, RSNA, COVID-19 RD	3	93.44	/
DeepCCXR [25]	CIDC	3	85	87
	COVID-19 Radiography Database	3	90	92
	9 datasets	3	93	94
**Our Model (MDCXR3-Net)**	**CIDC, COVID-19 Radiography Database**	**3**	**97.74**	**97.79**

In terms of the strengths and weaknesses of each model, we can see that the DeepCovidNet, CapsNet, and VGG16 models have lower accuracy and sensitivity compared to other models. This may indicate that their architectures are not optimal for this task or that their datasets may be limited in size and quality. The CoroNet and VGG19 models perform well, achieving high accuracy and sensitivity, but they use more complex architectures than some of the other models. The COVIDiagnosis-Net and CVDNet models also perform well with high accuracy and sensitivity, but they were trained on smaller datasets than some of the other models. It is also noteworthy that some models were trained on different datasets, which could explain the variability in their performance.

Furthermore, it can be noted that there is a correlation between the MDCXR3-Net model and the VGG16-based model proposed by Apostolopoulos and Mpesiana [24]. Methodically, both models used transfer learning with VGG16 as the backbone network. However, our proposed model, MDCXR3-Net, used a modified architecture with additional convolutional layers and dropout regularization to improve the model performance. On the other hand, the VGG16-based model proposed by Apostolopoulos and Mpesiana used the original architecture of VGG16 with a few modifications in the fully connected layers. Indeed, MDCXR3-Net achieved a higher disease detection accuracy and sensitivity compared to the VGG16-based model [24] which achieves an accuracy of 83.5 % and a sensitivity of 94.43 %. This is due to the modified architecture of MDCXR3-Net, which includes additional convolutional layers and dropout regularization, allowing the model to learn more complex features and reduce overfitting. Consequently, our proposed MDCXR3-Net-approved performance demonstrates its potential for accurate and sensitive COVID-19 and pneumonia detection, which will proficiently aid in their timely and effective diagnosis. However, there is still room for improvement in terms of optimizing architectures and testing on larger and more diverse datasets.

Besides, Table 8 displays a comparison of the MDCXR4-Net proposed model accuracy with other works from the literature that deal with the four classes of pulmonary infections (COVID-19, pneumonia, pulmonary opacity, and normal lung). The dataset used in each study is also mentioned in the table. The performance of a model depends on many factors including the quality and quantity of the data, the preprocessing techniques used, the choice of hyperparameters, and the architecture of the model itself. Therefore, it is challenging to make a direct comparison between different models on different datasets. Indeed, the presented results in Table 8 attest that the MDCXR4-Net model is highly effective in detecting the different types of pulmonary diseases. It has outperformed all the reported models (ResNet-50, DenseNet-121, Inception-v3, MobileNet-v2, and CoroNet) in terms of accuracy, and sensitivity by achieving an accuracy of 91.22 % and a sensitivity of 100 %.

**Table 8:** Performance comparison of MDCXR4-Net model with similar works.

Model	Dataset	# Classes	Accuracy (%)	Sensitivity (%)
ResNet-50 [64]	HSCX dataset	4	85.60	97.30
Chexnet: based DenseNet-121 [54]	ChestX-ray8 dataset	4	80.40	94.50
DeepCNN Based Inception-v3 [20]	MIMI-CXR dataset	4	78.80	89.70
MobileNet-v2 [15]	ChestX-ray14 dataset	4	86.6	98.4
CoroNet [17]	CIDC, RSNA	4	89.6	96.4
**MDCXR4-Net**	**CIDC, COVID-19 Radiography Database**	**4**	**91.22**	**100**

It is important to note that the performance of these models is highly dependent on the quality and size of the dataset used for training and testing. In this regard, our proposed model achieved the highest accuracy despite using a relatively small dataset, which is a notable strength of our approach. In addition, MDCXR4-Net was designed to be a lightweight model with fewer parameters compared to other models, which makes it more suitable for deployment on resource-limited devices such as mobile phones or embedded systems. Therefore, the choice of the model depends on the specific requirements of the application. Moreover, the proposed model has an advantage in its methodology, in that it does not use any data augmentation and preprocessing and we have not used any optimizer (for example: Nadam optimizer and SGD optimizer) to enhance the obtained results. Which can be considered a more challenging and realistic scenario. This makes the proposed model more robust and reliable for real-world applications.

Another consideration is the architecture of the models. Each model uses a different deep learning architecture, which may affect their performance. MDCXR4-Net uses a modified version of the VGG-16 architecture, while ResNet-50 uses a residual network, DenseNet-121 uses a dense network, and Inception-v3 uses an inception network. Each architecture has its strengths and weaknesses, which may affect the performance of the models.

Our proposed model has a unique architecture compared to the other models in the table, which allows it to capture the features specific to the different pulmonary disease classes. In addition, the results confirm that deep learning models are highly effective in diagnosing different types of pulmonary diseases. The proposed MDCXR4-Net model demonstrates superior performance compared to other state-of-the-art models. However, further studies are needed to explore the potential areas for improvement and to evaluate the performance of this model on larger and more diverse datasets. Indeed, it is worth noting that the performance of deep learning models can be further improved by optimizing hyperparameters and using advanced techniques such as data augmentation, transfer learning, ensembling, and deep learning optimizers. Therefore, there is always room for improvement and further research in this area.

Overall, compared to the reported methods from the literature, the proposed models are simpler with fewer parameters, accurate, and specific to the task in question. In addition, our models leverage the power of the VGG-17 architectures while tuned and fitted to achieve the highest classification performances making it a computationally efficient and practical solution for diagnosing pulmonary diseases using X-ray images. However, due to the lack of computational resources, our models have been evaluated on a relatively small sized dataset. So, testing on larger and more diverse datasets will support the model power. Moreover, it should be noted that more potential improvements can be investigated. In fact, due to the low quality of some dataset images, quality enhancement techniques may be conducted. In addition, data augmentation techniques (rotation, zooming, flipping, etc.) can be applied to increase the dataset size and help in boosting the model learning. Additionally, more diverse datasets such as the COVID-19 dataset [44,24] and the RSNA COVID-19 dataset [16,44,23,46] that include images from different populations and medical conditions may improve the generalizability of the models. Furthermore, other deep learning architectures and techniques may be explored to improve our models. Overall, our proposed models provide simple yet effective solutions for diagnosing pulmonary diseases using X-ray images. They outperformed most existing state-of-the-art methods and have the potential to improve the accuracy and efficiency of pulmonary diagnosis in clinical settings. Indeed, further studies are needed to fully validate their effectiveness and explore potential areas for improvement.

## 5 Conclusions

In this study, we introduced three innovative classification and diagnosis models for pulmonary diseases utilizing CXR radiological images of the lung. Our research showcased the remarkable efficacy of deep learning methodologies in accurately identifying various pulmonary infections, thereby providing invaluable support to clinicians and radiologists in the diagnostic process. Each of our proposed models was meticulously crafted on the VGG-16 architecture, with meticulous adjustments tailored to optimize performance for the specific classification tasks at hand. The first model achieved an exceptional accuracy of 99.09 % and a sensitivity of 99.13 % in detecting COVID-19 cases. Meanwhile, the second model, designed to distinguish between three classes of pulmonary infections (COVID-19, pneumonia, and normal lung), exhibited an impressive accuracy of 97.74 % with a sensitivity of 97.79 %. Lastly, our third model proficiently classified four distinct lung disease categories (COVID-19, pneumonia, pulmonary opacity, and normal lung) with an accuracy of 91.22 % and a sensitivity of 100 %.

Our models demonstrated robust performance across diverse datasets, surpassing the capabilities of many existing methods reported in the literature. Nonetheless, we recognize several areas ripe for enhancement. First and foremost, our models were evaluated on relatively small datasets, potentially constraining their applicability to broader and more diverse populations. Additionally, resource constraints during training and evaluation may have impeded scalability and efficiency. The inherent complexity of pulmonary diseases, compounded by variations in image quality, poses challenges in capturing nuanced features, thus impacting model performance. Moreover, ensuring the generalizability of our models across different populations, healthcare settings, and imaging protocols warrants further scrutiny and validation.

Looking ahead, there are promising avenues for advancing our research. Integrating additional deep learning models, such as ResNet or EfficientNet, using ensemble machine learning techniques could enhance classification accuracy further. Exploring the synergistic benefits of multimodal imaging data, including CT scans alongside CXR images, holds promise for augmenting disease detection accuracy. Furthermore, delving into transfer learning methodologies, where models leverage pre-training on large datasets and fine-tuning on smaller target datasets, may bolster accuracy in scenarios with limited data availability. Extending the scope of our models to encompass other medical domains, such as liver and brain disease diagnosis at early stages, could broaden their utility in healthcare applications.

These prospective avenues have the potential to catalyze significant advancements in the realm of deep learning-based disease detection, ushering in new paradigms of research and fostering improvements in healthcare outcomes. In summary, our proposed models represent a promising step forward in the realm of accurate and efficient pulmonary disease diagnosis using CXR images, laying the groundwork for transformative progress in medical imaging analysis.

## Data availability statement

Data will be made available on request.

## CRediT authorship contribution statement

**Akram Bennour:** Writing – original draft, Supervision, Data curation, Conceptualization. **Najib Ben Aoun:** Writing – review & editing, Methodology, Investigation, Formal analysis. **Osamah Ibrahim Khalaf:** Writing – review & editing, Validation, Supervision, Resources, Funding acquisition. **Fahad Ghabban:** Writing – review & editing, Visualization, Supervision, Methodology, Conceptualization. **Wing-Keung Wong:** Writing – original draft, Software, Methodology, Formal analysis. **Sameer Algburi:** Writing – original draft, Project administration, Methodology, Data curation.

## Declaration of competing interest

The authors declare that they have no known competing financial interests or personal relationships that could have appeared to influence the work reported in this paper.
